# Gut Microbiota Composition and Its Metabolites in Different Stages of Chronic Kidney Disease

**DOI:** 10.3390/jcm10173881

**Published:** 2021-08-29

**Authors:** Tso-Hsiao Chen, Chao-Wei Liu, Yi-Hsien Ho, Chun-Kai Huang, Ching-Sheng Hung, Barry H. Smith, Jung-Chun Lin

**Affiliations:** 1 Division of Nephrology, Wan Fang Hospital, Taipei Medical University, Taipei 116, Taiwan; 88128@w.tmu.edu.tw; 2 Department of Medicine, College of Medicine, Taipei Medical University, Taipei 110, Taiwan; 3 Ph.D. Program in Medical Biotechnology, College of Medical Science and Technology, Taipei Medical University, Taipei 110, Taiwan; rickliu1029@yahoo.com.tw (C.-W.L.); oryx@w.tmu.edu.tw (C.-S.H.); 4 Department of Laboratory Medicine, National Taiwan University Hospital, Taipei 100, Taiwan; 5 Department of Laboratory Medicine, Wan Fang Hospital, Taipei Medical University, Taipei 116, Taiwan; 94445@w.tmu.edu.tw (Y.-H.H.); 99421@w.tmu.edu.tw (C.-K.H.); 6 School of Medical Laboratory Science and Biotechnology, College of Medical Science and Technology, Taipei Medical University, Taipei 110, Taiwan; 7 Applied Medical Research Inc., Nashville, TN 37219, USA; Barry.Smith@dciinc.org; 8 Pulmonary Research Center, Wan Fang Hospital, Taipei Medical University, Taipei 116, Taiwan

**Keywords:** chronic kidney disease, fecal metabolite, gut microbiota, long-read sequencing, UPLC-coupled MS/MS

## Abstract

A growing body of study have documented the association of gut dysbiosis or fecal metabolites with chronic kidney disease (CKD). However, it is not clear whether the phenomenon simply reflects the microenvironment changes correlated with the CKD severity or contributes to the progression of CKD. In this study, we identified the gut microbiota and metabolite in feces samples correlated with CKD severity using the Nanopore long-read sequencing platform and UPLC-coupled MS/MS approach. A cross-sectional cohort study was performed from 1 June 2020 to 31 December 2020. One hundred and fifty-six clinical participants, including 60 healthy enrollees and 96 Stage 1–5 CKD patients, were enrolled in this study. The ROC curve generated with the relative abundance of Klebsiella pneumonia or S-Adenosylhomocysteine showed a gradual increase with the CKD severity. Our results further revealed the positive correlation of increased K. pneumonia and S-Adenosylhomocysteine in gut environment, which may be of etiological importance to the deterioration of a CKD patient. In that sense, the microbiota or metabolite changes constitute potential candidates for evaluating the progression of CKD.

## 1. Introduction

Irreversible pathological changes in renal function characterize chronic kidney disease (CKD), the third most prevalent chronic disease worldwide and affects an estimated 850 million people worldwide [1]. Hypertension, obesity, and diabetes mellitus (DM) are documented as critical risk factors for the development of CKD, which can, in turn, lead to end-stage renal disease (ESRD or kidney failure) [2]. Kidney failure, in turn, requires therapeutic interventions, including peritoneal and hemodialysis, and kidney transplantation, with the most common of these an expensive financial burden for the healthcare system. Accordingly, the prevention of CKD altogether and/or the slowing of its progression to end-stage disease must be a high priority [3].

The relevance between the gut bacterial community (also referred as the microbiota) and diverse diseases is an emerging avenue to pursue [4]. A growing body of evidence suggests the potential influence of CKD-associated gut dysbiosis on the progression of CKD through the gut-kidney axis [5,6]. Microbiota-population differentials of more than 100 operational taxonomic units (OTUs) have been identified using an animal kidney-failure model and in ESRD patients, compared to those in healthy counterparts [7,8]. For instance, decreases in the distribution of *Firmicutes*, *Actinobacteria*, and *Proteobacteria* with concomitant increases in *Bifidobacteria* and *Lactobacilli* were identified in ESRD patients [9,10]. A low-protein diet is commonly recommended to lessen the proteinuria with reduced intraglomerular pressure, sodium loading, and nitrogenous waste in moderate to advanced chronic kidney disease (CKD) patients [11]. Low-protein intake consequently led to gut dysbiosis and interfered with the gut microbiota-mediated fermentation, resulting in metabolic alteration with the generation of diverse metabolites [12,13]. A decrease in p-cresyl sulfate in serum with concomitant increases in *Blautia*, *Faecalibacterium*, and *Roseburia* species was identified in CKD patients receiving protein restriction [14]. Nevertheless, the impact of a low-protein diet on preserving the kidney function is continuously controversial and under-investigated [15]. Fiber supplementation was reported to be associated with an elevation in the relative abundance of *Faecalibacterium*, subsequently leading to the reduced systemic inflammation noted in ESRD patients [16]. In contrast, CKD-patient fecal microbiota transplantation to the gut of healthy mice resulted in insulin resistance, impaired kidney function, and uremia [17]. Nevertheless, the impact of gut dysbiosis or altered metabolite profile on the causation, progression, or gastrointestinal-environment toward CKD should be further clarified.

In this study, the gut dysbiosis or fecal metabolite profile in CKD patients in each of three stages and those of healthy participants was classified using long-read sequencing and LC-QTOFMS platform. Changes in the abundance of identified operational taxonomic unit (OUT) or metabolite in feces samples were evaluated in relation to CKD severity. The results of dual-omics assays suggested that the *K. pneumonia* and S-Adenosylhomocysteine functioned as the potential factors toward the causation or deterioration of CKD via gut-kidney axis.

## 2. Materials and Methods

### 2.1. Ethics Statement of the Study Cohort and Sample Collection

Enrollment of clinical participants and the experiments involving human participants were conducted according to the guidelines of the Declaration of Helsinki and approved by the Institutional Review Board of Taipei Medical University (approval no. N202003133). Formal informed consent was collected from the recruited participants prior to following experiment. Patients with CKD were enrolled from the Division of Nephrology at Taipei Municipal WanFang Hospital, and healthy participants were recruited from the Health Examination Center at Taipei Municipal WanFang Hospital. The stage of the CKD patient was defined as an estimated glomerular filtration rate (eGFR; mL/min/1.73 m^2^) in accordance with 2012 KDIGO clinical practice guideline for the evaluation and management of chronic kidney disease [18]. Accordingly, enrolled patients were grouped into mild (Stage 1: eGFR ≥ 90; Stage 2: eGFR 60–89), moderate (Stage 3A: eGFR 45–59; Stage 3B: eGFR 30–44), and advanced (Stage 4: eGFR 15–29; Stage 5 eGFR < 15) CKD. A comprehensive physical examination was conducted on all normal counterparts to ensure a satisfactory health status with particular respect to kidney function, diabetes, and hypertension. The recruitment of CKD patient was applied as well in another study [19]. A standard questionnaire was used to evaluate lifestyle, including diet, smoking, consumption of alcohol, and level of exercise of all participants. Use of antibiotics, a history of malignant disease, chemotherapy or radiation therapy, and regular use of a stool softener within the three months preceding entry into the study were all exclusion criteria.

### 2.2. Metadata and Biochemical Analysis

Peripheral venous blood samples were collected for serum creatinine (sCre) and fasting blood glucose (FBG) and assayed using Beckman Coulter AU5800 biochemical analyzers (Beckman Coulter Inc., Brea, CA, USA). Glycated hemoglobin (HbA1c) levels were determined using a Capillary 3 tera Instrument (Sebia, Lisses, France). Levels of fasting blood glucose and the percentage of glycated hemoglobin (HbA1c) in CKD patients were included to evaluate possible confounding variables related to diabetes. According to the contents of the questionnaire, participants were queried regarding age, gender, and dietary habits, including nutrient intake and any use of probiotics.

### 2.3. Bacterial DNA Extraction

Fecal samples were properly collected and preserved using a feces catcher and DNA/RNA Shield Fecal Collection tubes (Zymo Research, Irvine, CA, USA). Total genomic DNAs were extracted from 0.2 g of feces using the Quick-DNA Fecal/Soil Microbe Microprep Kit (Zymo Research) according to the manufacturer’s instructions. The quantity of extracted DNA was measured using a fluorometric kit (GeneCopoeia, Rockville, MD, USA) and a Qubit fluorometer (ThermoFisher Scientific, Wilmington, DE, USA). Qualified DNA samples were kept in a −80 °C freezer for subsequent DNA-sequencing analysis.

### 2.4. 16S Ribosomal (r)RNA Gene Sequencing

Bacterial subpopulations in fecal samples were analyzed using a third-generation long read-sequencing approach. In brief, 10 ng of extracted genomic DNA was subjected to 16S rRNA library construction using a Barcoding kit (SQK-16S024, Oxford Nanopore Technologies (ONT), Oxford, UK) according to the manufacturer’s protocol. The barcoded library was captured, washed, and eluted from magnetic beads (AMPure XP, Beckman Coulter, High Wycombe, UK). Two nanograms of the individual barcoded library were pooled, loaded, and sequenced on MinION flow cells (FLO-MIN106D R9.4.1, MinION instrument; Oxford Nanopore Technologies, Oxford, U.K.). The average length of sequenced read was 1540 nt, and the sequenced read number of each individual sample was 100,000 per sample to meet a sequencing depth of 50.

### 2.5. Metabolites Extraction

Fifty milligrams of sample was weighted to a micro-centrifuge tube, and 1 mL extract solution (acetonitrile:methanol:water = 2:2:1) was added. After 30 s vortex, the samples were homogenized for 4 min and sonicated for 5 min on ice. Then the samples were incubated for 1 h at −20 °C and centrifuged at 12,000 rpm for 15 min at 4 °C. The resulting supernatant was transferred to a fresh glass vial for analysis.

### 2.6. UPLC-MS/MS Analysis

Each sample (10 μL) was injected into a vanquish focused ultra-high-performance liquid chromatography (UHPLC) system coupled with an Orbitrap Elite Mass Spectrometry (Thermo Fisher Scientific) using electrospray ionization. UHPLC parameters were set as below: A 2.1 × 100 m² Acquity HSS T3 1.8 μm C18 column (Waters) was used. The column oven temperature was set at 40 °C. The binary mobile phase including deionized water containing 0.1% formic acid as solvent A, and LC-MS grade acetonitrile with 0.1% formic acid as solvent B. The flow rate was 0.25 mL/min with a linear gradient elution over 15 min. For the first minutes, solvent B percentage was held at 5%, linearly increased to 100% for the next 7 min, and kept constant for 3 min, then finally return to 5% in 1 min. To avoid any carry over effect, there was one blank injection after every sample injection, and one QC injection after every five sample injections for the peak area normalization. Mass spectrometry data were collected in positive mode with a default data-dependent acquisition method, one MS full scan performed in profile mode at 60,000 esolution, followed by 10 data-dependent MS2 scans at 15,000 resolution. The mass scan range was set from 70 to 1000 m/z. The normalized collision energy (NCE) of 25. The spray voltage was 3.5 kV, the capillary temperature was set at 280 °C. The sheath gas was set at 30 arbitrary units and the aux gas was set at five arbitrary units.

### 2.7. Bioinformatic Analysis

For gut microbiota analysis, the MinION-sequenced reads were first uploaded via the EPI2ME desktop agent (ONT) to the EPI2ME website algorithm (https://epi2me.nanoporetech.com, accessed on 1 May 2021). The quality and quantity of sequencing results were accessed through a web-interface. Analytical results of 16S rRNA classification were aligned for identification with the NCBI database, which contains 18,927 16S rRNA referents, using EPI2ME Labs Launcher (ONT). In brief, the 16S CSV file generated by EPI2ME Agent was subjected to alignment with NCBI taxonomy database through taxonkit software (v0.8.0, National Center for Biotechnology Information, Bethesda MD, USA). Subsequently, the counts of sequenced reads were extracted from the annotated table. Alpha- and beta-diversities of taxonomic profiling of MinION data were synchronously assessed using the Microbial Genomics Module (CLC genomics workbench (Qiagen v21.0.3; CLC Bio, Aarhus, Denmark)) with the 16S rRNA reference curated from the NCBI database.

### 2.8. UP-LC-MS/MS Data Preprocessing and Annotation

The raw data were converted to the mzXML format using ProteoWizard and processed with an in-house program, which was developed using R and based on XCMS, for peak detection, extraction, alignment, and integration. Then an in-house MS2 database (BiotreeDB V2.1; accessed on 1 June 2021) was applied in metabolite annotation. The cutoff for annotation was set at 0.3.

### 2.9. Statistical Analysis

Statistics regarding long-read sequencing results, including the number of total reads, read quality, and sequencing depth obtained by MinION sequencing, are shown as the mean ± standard error of the mean (SEM). Continuous variables were compared using a one-way analysis of variance (ANOVA), followed by Tukey’s multiple-comparison post-hoc test. A variable was considered to be significant with a *p* value of <0.05 (* *p* < 0.05; ** *p* < 0.01; *** *p* < 0.005). The availability of 50 participants in each group was sufficient to achieve a moderate effect size (0.60–0.08) with a significance of 5% and statistical power of 80% [20]. Differential abundances of the identified OTUs to the species level between the healthy group and CKD patients were assessed using a linear discriminant analysis effect size (LEfSe) assay through a website interface (https://huttenhower.sph.harvard.edu/galaxy/root, accessed on 4 May 2021) using default settings. The populations of identified gut OTUs between the healthy group and CKD patients were considered to differ statistically significantly with a linear discriminant analysis (LDA) score (log10) of >3 and a *p* value of <0.05. The utility of LDA-confirmed OTUs for predicting the occurrence of CKD was evaluated using the receiver operating characteristic (ROC) curve and area under the ROC curve (AUC) ratio as implemented in R programming. The correlation between the sub-populations of identified OTUs and clinical metadata was evaluated using the Spearman’s correlation coefficient.

## 3. Results

### 3.1. Demographic Data of Recruited Participants in This Study

**Figure 1:**
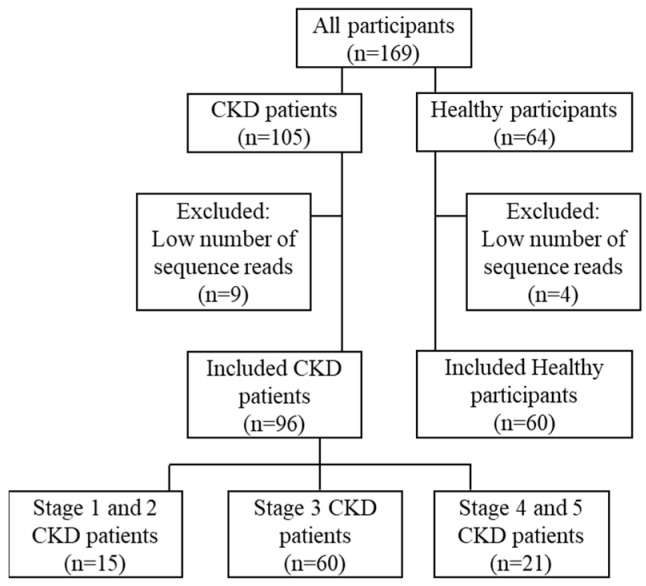
Flow chart demonstrates the enrollment of study participants.

To evaluate the correlation of the identities and distribution of the gut bacterial community sub-populations with CKD progression, 60 healthy participants and 96 patients, comprising 15 with stage 1–2, 60 with stage 3, and 21 with stage 4–5 CKD, were selected through a careful quality control for this study (Figure 1). As shown in Table 1, no difference in age or gender was observed among any of the groups. The levels of serum creatinine and the eGFR mirrored kidney-disease severity in the CKD patients.

### 3.2. Statistical Results of Gut Microbial Communities in Enrolled Subjected Assessed with Long-Read Sequencing Results

The average numbers of sequenced and qualified reads per sample were filtered and generated using the CLC Genomics Workbench (v.21.0.2, Aarhus, Denmark) (Table 2). As shown in Table 2, no significant differences in sequencing efficiency were noted among all groups.

**Figure 2:**
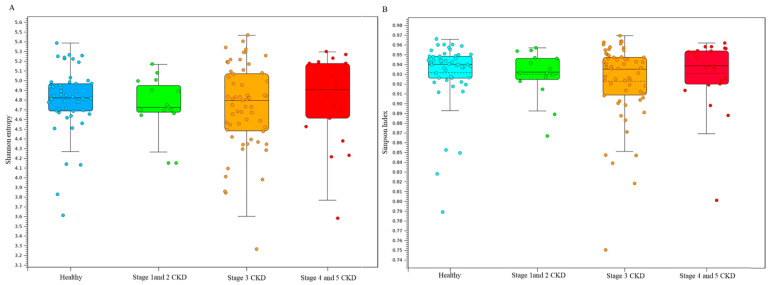
Diversity of taxonomic alignments between groups evaluated with long-read sequencing results. The alpha-diversity in all groups from the MinION results is illustrated using (**A**) Shannon entropy and (**B**) the Simpson index.

**Figure 3:**
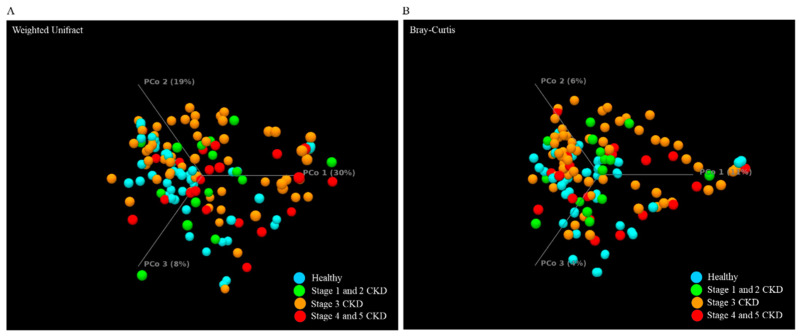
The dissimilarity between all groups with MinION sequencing results is illustrated using (**A**) Weighted Unifrac principal component analysis (PCoA) and (**B**) Bray–Curtis dissimilarity analysis.

Analyses using the Shannon entropy (Figure 2A) and Simpson indices (Figure 2B) showed no obvious difference in terms of α-diversity between the groups’ microbial communities from long-read sequencing results. The dissimilarity between the groups’ microbial communities was evaluated with the weighted Unifract distance or Bray–Curtis index. Statistical results of the weighted UniFrac or Bray–Curtis dissimilarity analysis principal coordinate analysis (PCoA) indicated that unique bacterial population aggregates were identified in fecal samples of CKD patient at distinct stages compared to those of the healthy group (Figure 3A,B). These results delineated differences in the composition rather than the richness or numerical abundance of the gut microbial communities between the CKD patients and healthy participants.

### 3.3. Identification and Comparison of the Microbial Communities in the Guts of the Healthy Group and CKD Patients Classified Using a Long-Read Sequencing Platform

**Figure 4:**
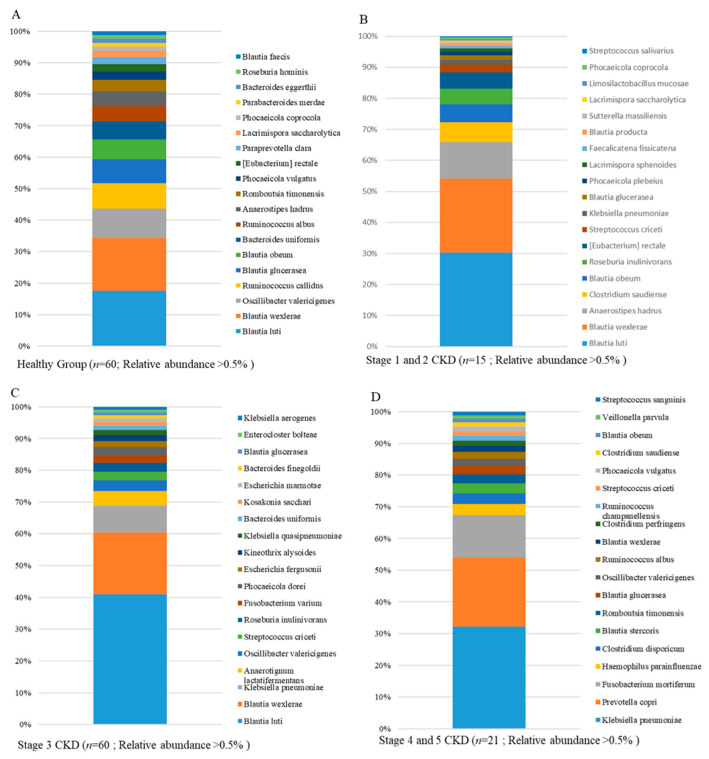
Characterization of identified taxa in healthy participants and chronic kidney disease (CKD) patients with long-read sequencing results. Relative abundances of the top 20 classified operational taxonomic units (OTUs) to species level in (**A**) the healthy group, (**B**) Stage 1 and 2, (**C**) Stage 3, and (**D**) Stage 4 and 5 CKD patients are shown on a stacked bar chart. Abundances of OTUs relative to all microbial communities exceed 0.5%.

**Figure 5:**
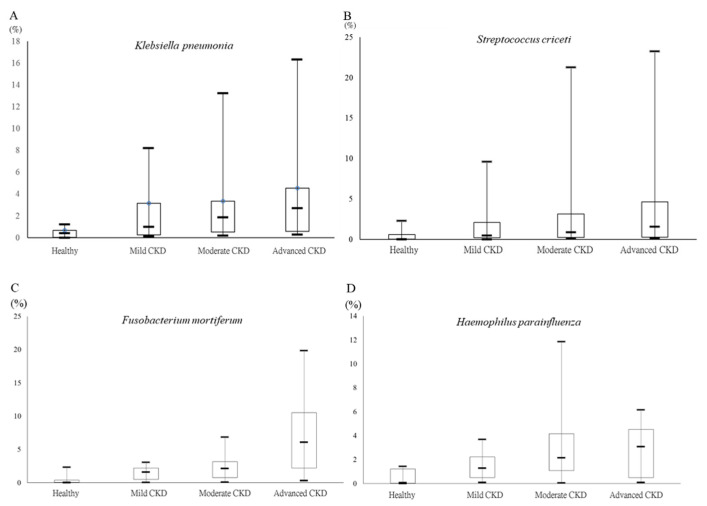
Differential abundances of identified taxa between healthy participants and chronic kidney disease (CKD) patients cross distinct stages. Relative abundances of (**A**) *Klebsiella pneumonia*, (**B**) *Streptococcus criceti*, (**C**) *Haemophilus parainfluenzae*, and (**D**) *Fusobacterium mortiferum* in the fecal samples of all groups are shown in a box plot.

The long-read sequencing approach has been demonstrated to exhibit a higher efficiency than that of short-read sequencing for taxonomic classification of the gut microbiota at the species level [21,22]. In this study, around 350–400 OTUs at the species level were classified in individual groups using MinION sequencing-coupled with the EPI2ME algorithm, and the top 20 classified OTUs at the species level in all groups are shown in Figure 3. The majority of the top-20 classified OTUs in the healthy group were normal gut flora, including the genera *Blautia*, *Anaerostipes*, *Bacteroides*, and *Ruminococcus* (Figure 4A). Increases in the relative levels of genera *Streptococcus*, *Klebsiella pneumonia*, and *Haemophilus parainfluenzae* were identified in the gut microbiota of patients with CKD at distinct stages (Figure 4B–D). The relatively higher abundances of *Fusobacterium varium* or *Fusobacterium mortiferum* were classified in fecal samples of stage 3 CKD patients (Figure 4C) or stage 4 and 5 CKD patients (Figure 4D). Gradual increases in the relative abundances of *K. pneumonia*, *S. criceti*, and *H. parainfluenzae* were further identified at distinct stages of CKD as compared to the healthy group (Figure 5A–C). A significant elevation in the relative level of *F. mortiferum* was solely noted in feces samples of stage 4 and 5 CKD patients, compared to the other groups (Figure 5D). These results indicate the presence of opportunistic pathogens in the gut microbiota of patients across distinct CKD stages.

### 3.4. Differential Abundances of Identified OTUs at the Species Level between Healthy Participants and CKD Patients Evaluated Statistically

**Figure 6:**
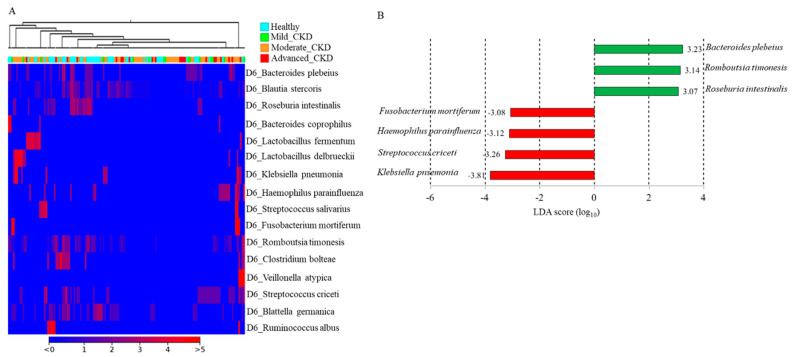
Identified OTU at the species level differed in the abundances between healthy participants and chronic kidney disease (CKD) patients. (**A**) Correlations between the differential abundances of the top 16 classified OTUs at the species level and all recruited participants are shown in a heat map chart. (**B**) Histogram of linear discriminant analysis (LDA) scores computed for OTUs with differential abundances in healthy participants (green bar) and CKD patients (red bar).

To assess the utility of long-read sequencing results, differential populations identities and quantities of the identified OTUs between the healthy group and CKD patients were evaluated through statistical analyses. A heat map illustrating the differential abundances of 16 identified OTUs was generated using the CLC Genomics Workbench (v.20.0.1). The increased levels of *K. pneumonia*, *H. parainfluenzae*, *F. mortiferum*, *Lactobacillus delbrueckii*, and *S. criceti* were noted in CKD patients across distinct stages compared to the healthy group (Figure 6A). Differential abundances of identified OTUs between healthy participants and CKD patients were further evaluated using a linear discriminant analysis (LDA) effect-size (LEfSe) assay [23]. The LDA score indicated relatively numerically high abundances of *K. pneumonia*, *S. criceti*, *H. parainfluenzae*, and *F. mortiferum* in the microbial communities of CKD participants (Figure 6B, red bars) compared to the healthy group (LDA score (log10) > 3). In contrast, *Bacteroides plebeius*, *Romboutsia timonesis*, and *Roseburia intestinalis* were relatively more abundant in the gut microbiota of healthy participants (Figure 5B, green bars) (>0.5%) compared to all other microbial communities.

### 3.5. Metabolic Profiles among Healthy Participants and CKD Patients across Distinct Stages

**Figure 7:**
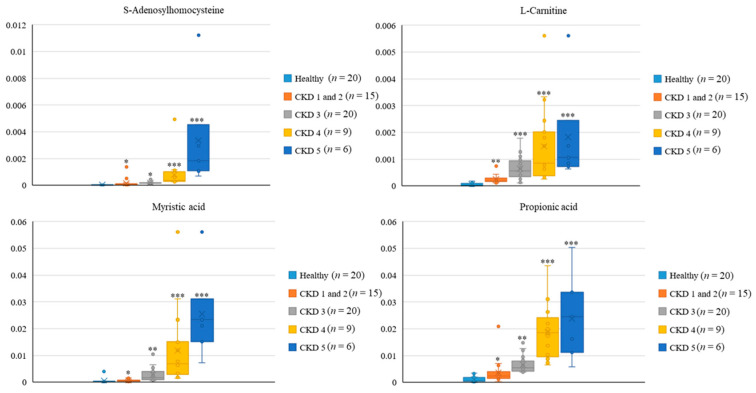
Severity-related changes in fecal metabolic signatures of CKD across distinct stages. Intensity of fecal metabolites among different groups were analyzed by Wilcoxon rank sum test. *** *p* < 0.001; ** *p* < 0.005; * *p* < 0.01.

To further validate the relevance of CKD severity with gut metabolite profile generated by host cells and gut microbiota, the feces samples collected from the healthy participants (*n* = 20), CKD 1 and 2 patients (*n* = 15), CKD 3 patients (*n* = 20), and CKD 4 and 5 patients (*n* = 15) were subjected to LC-QTOFMS analyses. The discriminating metabolites were subjected to following analysis with the criteria, including VIP (variable importance in projection) values > 1.5 and *p* < 0.05. As shown in Table 3, a total of 10 metabolites were selected in this study. As compared to the healthy controls, the significant increases in all identified metabolites (fold-change >2; *p* < 0.05) were noted in the feces samples of CKD patients (Table 3). The relative standard deviation (RSD) for the 10 differential metabolites varying from 3.37 to 24.62%, which suggested the analytic consistency throughout the whole study. Gradual increases in the discriminating intensity of four metabolites with high VIP value, including S-Adenosylhomocysteine, Propionic acid, Myristic acid, and L-Carnitine, were noted across the distinct stages of CKD (Figure 7). These results suggested the potential utility of gut metabolites on evaluating or predicting the severity of CKD.

### 3.6. Potential Utility of Identified OTUs or Gut Metabolites on Differentiating CKD Subjects across Distinct Stages

**Figure 8:**
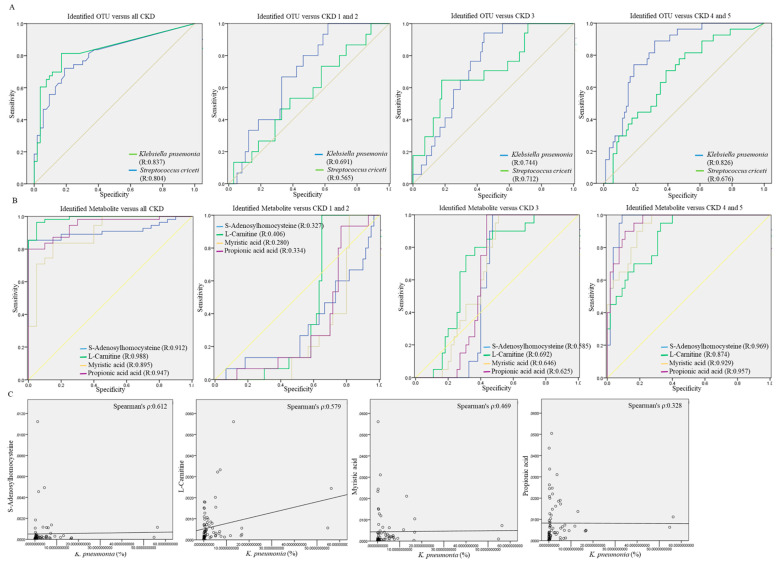
Predictive performances of identified OTUs or fecal metabolites toward the occurrence of chronic kidney disease (CKD) were evaluated using the random forests model. The area under the receiver operating characteristics (ROC) curve (AUC) was applied for differentiating all CKD patients or severity-specific CKD patients from the healthy group with (**A**) the relative abundances of identified OTUs or (**B**) the intensity of identified metabolites in fecal samples. (**C**) The correlation of severity-specific OTUs with the intensity of fecal metabolites was evaluated using the Spearman’s correlation coefficient.

To evaluate the potential of the gut microbiota in differentiating CKD patients from healthy participants, a random forest regression model was constructed with the differential abundances of identified OTUs using the receiver operating characteristics (ROC) curve. The ROC curve generated with the relative abundance of *K. pneumonia* or *S. criceti* toward the diagnosis of all CKD patients resulted in an area under the ROC curve (AUC) of 0.837 or 0.804 (Figure 8A, left panel). Increases in the AUCs generated with the quantitative populations of *K. pneumonia* or *S. criceti* with respect to the severity of CKD (Figure 7 middle and right) suggested the utility of identified OTUs on the diagnosis of patient with late-stage CKD.

By contrast, the higher AUC values generated with the intensity of four metabolites toward the diagnosis of all CKD patients suggested the greater utility that gut metabolites exerted than those of gut dysbiosis on diagnosis of all CKD patients (Figure 8B, left). Nevertheless, the predictive utility of four metabolites was much more efficient toward the stage 4 and 5 CKD patients as compared to other participants (Figure 8B, middle and right). Besides Propionic acid, the positive correlation of the relative abundance of *K. pneumonia* with S-Adenosylhomocysteine (Figure 8C, ρ = 0.612), L-Carnitine (Figure 8C, ρ = 0.579), and Myristic acid (Figure 8C, ρ = 0.469) were noted in the feces samples of CKD patients. These results suggest the usefulness of the increased levels of *K. pneumonia* or gut metabolites as potential candidates for evaluating the deterioration of patient with early-stage CKD.

## 4. Discussion

With advancements in high-throughput sequencing approaches, gut dysbiosis has been observed in diverse diseases and, therefore, suggested to be important in understanding these disease processes [24]. It is crucial to realize whether gut dysbiosis is predictive and/or causative of disease progression, or simply a passive biomarker of disease state. Herein, we conducted a cross-sectional cohort study to classify microbial communities at the species level, and its association with fecal metabolites across different stages of CKD. These results highlight a potential host-microbe-metabolite axis that was relevant to the severity of CKD.

The association of gut dysbiosis and changes of the intestinal wall or inflammatory activity has been observed in patients with diabetes-induced CKD [25,26]. The result of 16S rRNA sequencing demonstrated that less diversity in microbial community and dominance of opportunistic pathogenic taxa, including genera *Klebsiella* and *Enterobacteriaceae*, with concomitant decreases in the abundances of *Roseburia* and *Blautia* genera were found in the feces samples of patients with T2DM [27]. Hyperglycemia and gut dysbiosis may constitute a potential circuit increasing the permeability of intestinal wall and inflammatory activity with resulting in the initiation or progression of CKD. Fecal transplantation from healthy donors was reported to lessen the deterioration of diabetes patients with reprogrammed gut dysbiosis, suggesting the potential determinant that gut microbiota serve throughout the process of disease [28,29]. The integrity and barrier function of gut wall was compromised both in patients with CKD-related factor [26,30,31]. Changes in the richness of gut microbial genera related to the mucosal barrier function of intestinal wall, such as the decreases in *Roseburia* and *Faecalibacterium* with concomitant increases in *Clostridium perfringens*, *Betaproteobacteria*, and *Desulfovibrio*, were found in diabetes patients [32,33]. The richness of genera *Roseburia*, *Faecalibacterium*, or *Bifidobacteria* has been correlated with increased levels of butyrate [34,35], a short chain fatty acid, which strengthens the integrity of gut barrier through stable tight and efficient mucus production [36,37]. Moreover, a disrupted gut barrier was noted in individuals with increased abundance of *K. pneumonia* in gut microbiota [38]. Gut barrier dysfunction resulted in the leakage of pro-inflammatory product generated by the pathogenic taxa, subsequently leading to insulin resistance and progression of CKD in diabetic patients [39,40]. By using the long-read sequencing platform, an increase in the relative level of *K. pneumonia* in gut microbiota of CKD patient was consistently noted, which suggested the potential impact of *K. pneumonia* on the progression of CKD. In addition, the influence of colorectal cancer-related *F.mortiferum* on CKD development was worthy of further investigation.

Several gastrointestinal bacteria were recently demonstrated to be highly relevant to the elevation or generation of uremic toxins, which served the independent risk factor in CKD patient [41]. We identified the gradual increases in fecal S-adenosylhomocysteine, L-Carnitine, Propionic acid, and Myristic acid using untargeted LC-MS/MS platform across the progression of CKD, which was consistent with other previous reports [42,43,44,45]. Among these four metabolites, the positive relevance of renal dysfunction or insufficiency in ESRD or T2DM patients with the elevated S-adenosylhomocysteine level in serum or urine was frequently identified in previous studies [46,47]. Accumulation of S-adenosylhomocysteine consequently interfered with post-translational or epigenetic regulation involved in the activity of methylation reactions that were related to the renal function [48]. *K. pneumonia* has been demonstrated to be capable of encoding the 5’-methylthioadenosine/S-adenosylhomocysteine nucleosidase involved in the production of S-adenosylhomocysteine [49]. In addition, the presence of L-Carnitine was documented to facilitate the interaction between host cells and *K. pneumonia*, which may interfere with the integrity or barrier function of the intestinal wall [50]. These results suggested that the interplay between *K. pneumonia* and S-Adenosylhomocysteine may function as a putative mechanism toward the deterioration of CKD. Recruitment of single ethnicity population and uneven case number in each CKD group are the limitations in this study. Nevertheless, the influence of identified candidate on CKD progression might be deciphered by conducting a longitudinal study with the same study participants.

## 5. Conclusions

In conclusion, our findings facilitated a further understanding in the relevance between the gut environment and CKD across different stages. These results provided a potential avenue for emerging diagnosis or intervention of renal impairment with CKD-related microbiome or metabolite.

## Figures and Tables

**Table 1:** Demographics of healthy participants and enrolled chronic kidney disease patients.

	Healthy Group *(n* = 60*)*	Stage 1 and 2 CKD *(n* = 15*)*	Stage 3 CKD *(n* = 60*)*	Stage 4 and 5 CKD *(n* = 21*)*	*p* Value
Ethnicity (Taiwanese)	60 (100%)	15 (100%)	60 (100%)	21 (100%)	No Difference
Age (Median(IQR))	66 (41–87)	67 (50–84)	71 (33–90)	71 (43–87)	>0.5
Sex Female Male					>0.5
	32 (53.3%) 28 (46.7%)	4 (26.67%) 11 (73.33%)	30 (50%) 30 (50%)	10 (47.62%) 11 (52.38%)	
T2DM	0 (0%)	6 (40%)	9 (15%)	7 (33.3%)	>0.5
Fasting Blood Glucose (mg/dL) (Median(IQR))	89 (61–100)	114 (91–425)	101 (81–216)	108 (77–278)	>0.5
PleaseHbA1c (%) (Median(IQR))	5.1 (4.2–6.0)	7.05 (5.2–9.4)	5.8 (4.1–8.6)	6 (4.7–8.5)	>0.5
Serum Creatinine (mg/dL) (Median(IQR))	0.72 (0.5–1.15)	1.05 (0.73–1.41)	1.475 (0.86–3.36)	3.12 (1.96–13.15)	0.031
eGFR (ml/min/1.73 m^2^) (Median(IQR))	92.4 (63.9–134.2)	69.88 (63.57–81.18)	43.37 (37.32–51.7)	18.11 (10.04–25.73)	0.018

**Table 2:** Summary statistics of long-read sequencing results.

Group	Healthy Group *(n* = 60*)*	Stage 1 and 2 CKD *(n* = 15*)*	Stage 3 CKD *(n* = 60*)*	Stage 4 and 5 CKD *(n* = 21*)*	*p* Value
Number of Raw reads per sample	86,121 (±7321)	101,233 (±15,702)	91,775 (±13,488)	113,206 (±23,517)	>0.5
Number of qualified reads per sample	63,879 (±5121)	93,508 (±8332)	80,034 (±7540)	96,681 (±8116)	>0.5
Correctly classified (% (SD))	89.22 (5.64)	90.13 (4.97)	91.59 (5.06)	87.31 (7.21)	>0.5

**Table 3:** Summary statistics of top 10 discriminating metabolites in feces samples.

Metabolite	KEGG	HMDB	Microbe	Hsa	Healthy	CKD	VIP	*p* Value	Fold Change	RSD (%)
S-Adenosylhomocysteine	C00021	HMDB0000939	+	+	1.81 × 10^−5^	0.000258	2.51	0.029	6.94	3.37
Propionic acid	C00163	HMDB0000619	+	+	0.000239	0.012946	2.36	0.021	3.17	23.17
Myristic acid	C06424	HMDB0000806	+	+	0.005311	0.001116	2.35	0.019	3.49	9.85
L-Carnitine	C00318	HMDB0000062	NA	+	7.44 × 10^−6^	0.000509	2.25	0.131	2.72	19.38
Capsaicin	C06866	HMDB0002227	+	+	0.000343	0.002697	1.75	0.034	6.02	11.52
L-Tyrosine	C00082	HMDB0000158	+	+	0.000186	0.005451	1.74	0.037	4.81	24.62
Ephedrine	C01575	HMDB0015451	+	+	4.51 × 10^−6^	0.000865	2.03	0.019	6.25	17.22
gamma-Terpinene	C09900	HMDB0005806	NA	NA	0.000131	0.001716	1.39	0.039	4.43	20.15
Tricetin	C10192	HMDB0029620	NA	NA	0.120803	0.000461	1.27	0.124	3.09	20.54
Trehalose	C01083	HMDB0000975	+	+	0.000273	0.02608	1.21	0.042	6.18	24.15

## Data Availability

The data presented in this study are available on request from the corresponding author due to privacy restrictions.
